# The Evolution of the Management of Dysplasia in Ulcerative Colitis

**DOI:** 10.3390/cancers18071165

**Published:** 2026-04-04

**Authors:** Adrienne L. Vickers, Alessandro Fichera

**Affiliations:** 1 Department of Surgery, Weill Cornell Medicine, NewYork-Presbyterian, 1300 York Avenue, New York, NY 10065, USA; 2 Section of Colon and Rectal Surgery, Department of Surgery, Weill Cornell Medicine, NewYork-Presbyterian, 1300 York Avenue, New York, NY 10065, USA

**Keywords:** dysplasia, ulcerative colitis, colorectal cancer

## Abstract

With modern medicine and a better understanding of the ulcerative colitis disease process, there have been many changes in how we manage ulcerative colitis-related dysplasia over the past 20 years. One of the biggest concerns for these patients, given the inflammatory nature of their disease, is the progression from chronic inflammation to cancer. Patients with ulcerative colitis have about a 2.4-fold increased risk of developing colorectal cancer compared to the general population, which is concerning since colorectal cancer is the 2nd leading cause of cancer-related deaths in the United States. Traditionally, surgery was the preferred treatment for ulcerative colitis patients with dysplasia, but now, with advances in surveillance such as high-definition colonoscopy and chromoendoscopy, the management approach is more nuanced. Understanding the risk for different individuals within this patient population is key to comprehensive and personalized care management. In this review article, we will explore risk factors, surveillance methods, and classification.

## Simple Summary

With modern medicine and a better understanding of the ulcerative colitis disease process, there have been many changes in how we manage ulcerative colitis-related dysplasia over the past 20 years. Patients with ulcerative colitis have about a 2.4-fold increased risk of developing colorectal cancer compared to the general population, which is concerning since colorectal cancer is the 2nd leading cause of cancer-related deaths in the United States. Colonoscopy surveillance is the main means of identifying this progression, while the classification and management of dysplasia continue to change. The aim of this review is to summarize the risk factors, surveillance methods, classification, and current management guidelines for ulcerative colitis-related dysplasia.

## 1. Introduction

Ulcerative colitis is a chronic inflammatory disorder with relapsing and remitting mucosal inflammation and an increased risk of colorectal neoplasia. Twenty years ago, after a 45-year-old female with a history of left-sided ulcerative colitis (UC) was found to have unifocal visible dysplasia, she underwent a total proctocolectomy. At that time, surgical intervention was widely recommended due to concerns about synchronous neoplasia and the limitations of endoscopic detection techniques. However, with improvements in medical care, endoscopic imaging, and resection techniques, this patient can now be managed with endoscopic resection and followed with close surveillance. This clinical scenario highlights the evolution in the management of colitis-associated dysplasia and reflects a shift toward more individualized, risk-adapted care.

Colorectal cancer remains the most feared long-term complication of ulcerative colitis and is a significant contributor to morbidity and mortality in the US. It is the second most common cause of cancer-related deaths in the US and the third most diagnosed cancer [1,2]. Patients with long-standing UC have an estimated 2–3-fold increased risk of developing colorectal cancer (CRC) [3]. In an updated population-based systematic review and meta-analysis, the pooled standardized incidence ratio (SIR) was 2.48 (95% CI 1.64–3.76) compared to the general population [4]. The link between ulcerative colitis and colorectal cancer was first reported in a case report in 1925, with further research firmly establishing this connection [5]. Since then, the management of patients with ulcerative colitis with dysplasia has greatly evolved. Advances in surveillance, including high-definition (HD) colonoscopy and chromoendoscopy, have markedly improved dysplasia detection and characterization of lesions. These developments, combined with improvements in medical therapy, including the development of drugs such as biologics, have changed the management paradigm for colitis-associated dysplasia. Appropriately managing patients has become even more critical, as the global burden of ulcerative colitis continues to rise.

The geographic distribution of ulcerative colitis is expanding. Ulcerative colitis was once thought to be a disease primarily of Westernized countries. A systematic review that examined over 200 reports found that the highest prevalence and incidence of inflammatory bowel disease (IBD) are in Canada and Europe, with the US also high [6]. A more recent systematic review of 147 studies showed that, although prevalence has increased in the Western world, incidence has either plateaued or decreased [7]. Of the 15 studies that looked at UC, only one reported an increase in incidence in the Western world. The increased prevalence in the Western world is thought to be secondary to compounding prevalence, a cumulative effect of incident cases leading to an exponential increase in overall prevalence. This occurs in the setting of chronic disease with a low mortality and an early age of onset [8]. Despite IBD being more prevalent in the Western world, affecting up to 0.5% of the general population, it has now become a global disease in the 21st century, and its incidence continues to rise in countries such as those in Asia, South America, and the Middle East as they become more industrialized [7,8,9]. Exposure to potential environmental risks associated with the increasing industrialization and urbanization of certain regions is thought to be one of the reasons for the increase in the incidence of IBD. However, the precise mechanism and the relationship between the environment and genetic susceptibility in the development and pathogenesis of IBD are still largely unknown [6]. Overall, IBD is now considered a global disease with an increasing prevalence, making understanding the management and care of this patient population even more crucial. In this review article, we will briefly explore the pathogenesis, risk factors, detection/surveillance, classification, and current management guidelines for ulcerative colitis-related dysplasia.

## 2. Pathogenesis

**Figure 1:**
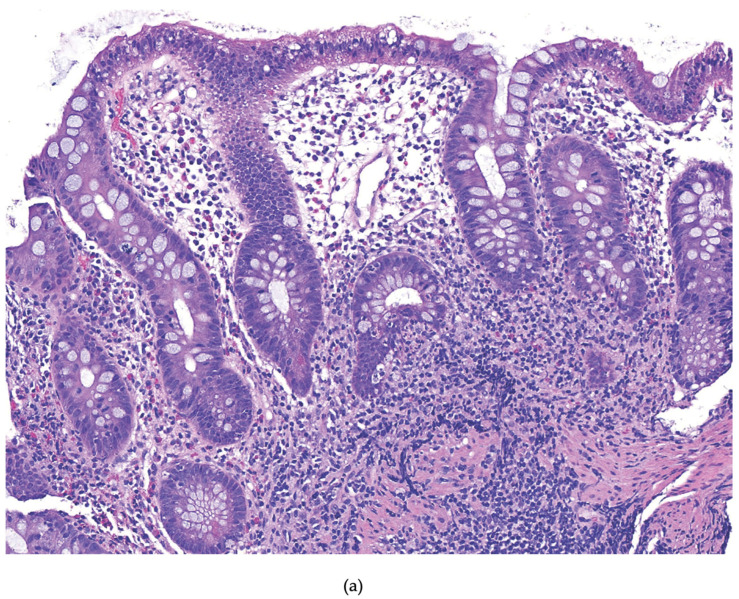

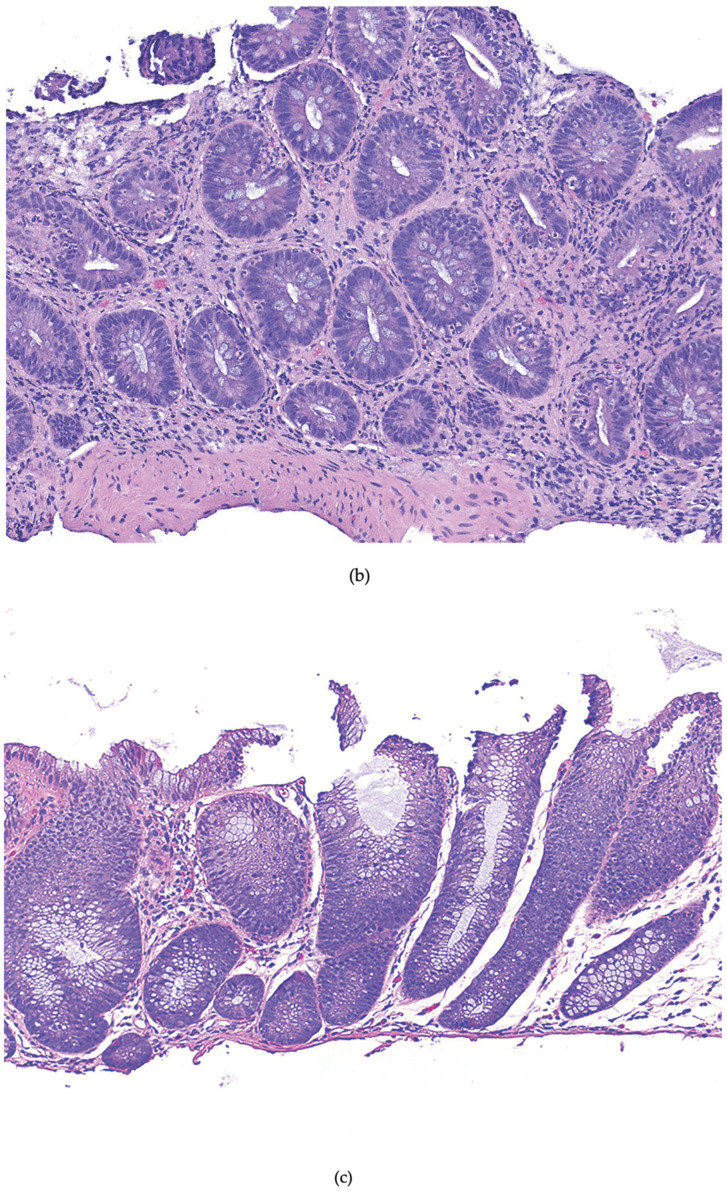

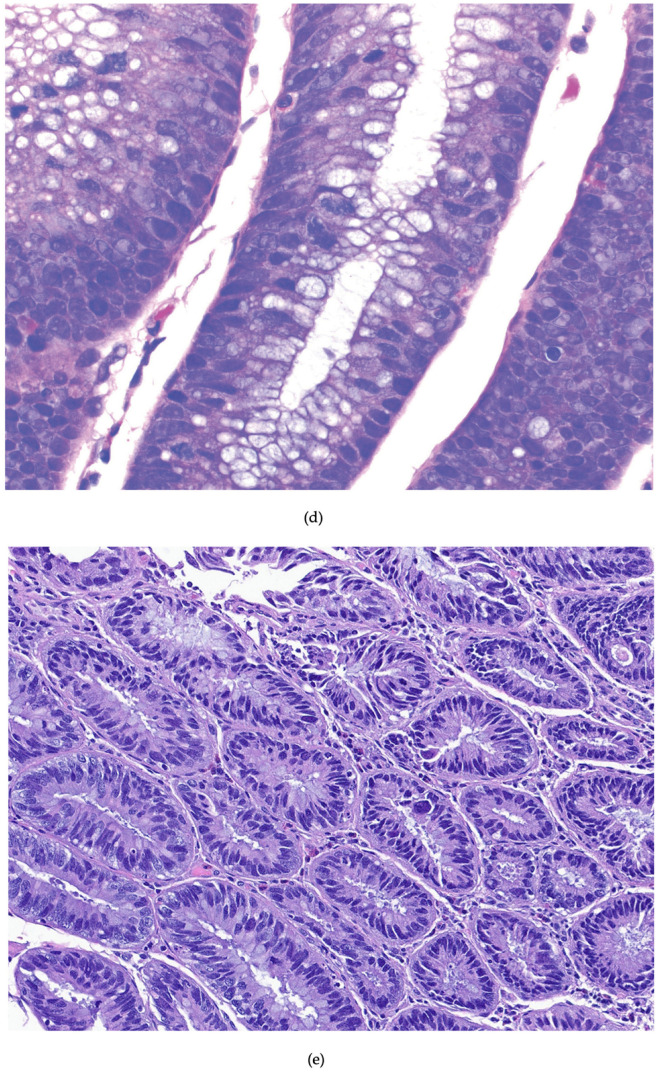
Inflammatory bowel disease-associated dysplasia spectrum. (**a**) IBD no dysplasia mucosa shows chronic active inflammation with basal lymphoplasmacytosis and neutrophilic cryptitis, but there is normal maturation of the crypts from base to surface; (**b**) IBD indefinite dysplasia cytologic features of the crypt epithelium are similar to those seen in LGD; however, the presence of neutrophilic cryptitis and absence of evaluable surface epithelium preclude definitive classification; (**c**) IBD LGD shows abnormal cell growth confined to the basement membrane of the native crypt architecture; (**d**) IBD LGD high power of previous image shows cells that show lack of maturation with increased nuclear to cytoplasmic ratio, but with maintained polarity; (**e**) IBD HGD shows an increased degree of nuclear atypia, loss of cell polarity, and frequent mitotic activity. Magnifications: (**a**) 100×; (**b**) 100×; (**c**) 100×; (**d**) 400×; (**e**) 200×.

The pathway from chronic colitis to colorectal cancer is complex and involves several factors, including the host immune system and inflammatory response. The histopathologic progression from colitis to dysplasia is generally described as follows: no dysplasia–to indefinite dysplasia–to low-grade dysplasia–to high-grade dysplasia–to carcinoma. However, this progression does not always follow a linear path to cancer, with some steps bypassed at first or not occurring at all [10]. Colitis-associated neoplasia (CAN) is postulated to arise from multiple dysplastic areas within the chronically inflamed colonic mucosa. This highlights the concept known as “field effect” or “field cancerization,” in which persistent inflammation leads to a series of molecular alterations that create extensive regions of mucosa at risk of neoplastic transformation [11,12,13]. This mechanism contrasts with sporadic CRC, which typically originates from one or two dysplastic foci [10,14]. Dysplasia refers to abnormal or disordered cell growth, indicating a precancerous change confined within the basement membrane. It can be broadly classified into no dysplasia, indefinite dysplasia, low-grade dysplasia, or high-grade dysplasia [14]. Each subset has specific cytologic and architectural features. Indefinite dysplasia refers to cytologic atypia that does not fit a definitive classification for any number of reasons, including background active inflammation. Low-grade dysplasia (LGD) is characterized by maintenance of cellular polarity and exhibits few typical mitotic figures. In comparison, high-grade dysplasia (HGD) has histologic features such as loss of nuclear polarity, high nuclear-to-cytoplasmic ratios, and atypical mitotic figures [15]. These histologic distinctions carry important clinical implications, as high-grade dysplasia is associated with a relatively higher risk of malignant transformation compared to low-grade dysplasia (Figure 1).

The hallmark of the colitis-associated dysplasia pathway is chronic, long-standing, low-grade inflammation, which is a driver of neoplastic transformation. Persistent mucosal inflammation activates an immune pathway that recruits inflammatory cells, creating a cycle that ultimately leads to changes in epithelial proliferation, survival, and migration [16]. More specifically, the persistent presence of inflammatory cells leads to the production of pro-inflammatory cytokines, such as interleukin-1, interleukin-6, and tumor necrosis factor alpha, as well as chemokines, thereby perpetuating epithelial injury. These mediators activate downstream transcription factors that sustain inflammation and promote carcinogenesis, mainly through the early loss of the p53 tumor suppressor gene and the activation of NF-kB and STAT3.

Genomic instability further contributes to carcinogenesis in colitis-associated neoplasia. Chromosomal instability and microsatellite instability represent the two most common somatic genetic abnormalities associated with CRC. They occur with similar frequency in CAN and sporadic CRC, but the timing of their occurrence differs [14,16]. The loss of p53 usually occurs later in sporadic CRC carcinogenesis than in colitis-associated cancer. Conversely, the loss of adenomatous polyposis coli (APC) function typically occurs earlier and more often in sporadic CRC development than in colitis-associated cancer [10,14]. Ultimately, repeated cycles of inflammation, epithelial injury, and epithelial regeneration in the presence of oxidative stress generate an increase in DNA damage and ultimately foster a microenvironment conducive to mucosal neoplastic transformation. The pathogenesis of colitis-associated neoplasia involves multiple factors that reflect a complex interplay among genetic susceptibility, environmental exposures, immune response, and the gut microbiome, with dysregulation of any component increasing the risk of developing colorectal cancer.

## 3. Risk Factors

Understanding the risk factors for colitis-associated dysplasia and CRC is essential for caring for patients with ulcerative colitis. The overall risk of CRC increases approximately 8–10 years after diagnosis, with the duration of chronic inflammation being a well-established independent risk factor [17]. A meta-analysis on UC revealed a cumulative cancer risk 2% at 10 years, increasing to 18% at 30 years [18]. A more recent meta-analysis focusing on IBD, not just UC, reported a cumulative cancer risk of 1% at 10 years of disease and 5% after more than 20 years [19]. This second meta-analysis showed a lower cumulative cancer risk, potentially due to an aging cohort in which high-risk patients were already censored prior to follow-up and a more homogeneous set of studies, leading to a more accurate estimation of CRC risk. The anatomical extent, location, and severity of UC are also important risk factors. Patients with pancolitis, left-sided disease, or more proximal disease are at higher risk for CRC [18,20]. However, patients with ulcerative proctitis have a low risk, with some studies finding it comparable to that of the general population [10,17]. Primary sclerosing cholangitis (PSC) is another significant risk factor, increasing the CRC risk and necessitating earlier and more frequent surveillance. A meta-analysis of 11 studies found a nearly fourfold increase in colorectal neoplasia among UC patients with PSC compared to those without [21]. Additional factors include a family history of sporadic CRC or UC, particularly in first-degree relatives, previous colorectal cancer, and age at disease onset [17,22]. While some risk factors are unmodifiable, others, such as cumulative inflammatory burden, can be reduced through advances in medical care and drugs like biologics. Research indicates a decline in CRC risk among UC patients, largely attributed to improved treatments that control inflammation and reduce disease severity and extent, as well as enhanced surveillance for precancerous lesions [20,23]. Recognizing a patient’s specific risk factors is important for individualized risk stratification when determining a management and surveillance plan that incorporates current guidelines and the ever-evolving endoscopic techniques and therapeutic options.

## 4. Surveillance

### 4.1. History and Evolution

Surveillance plays a crucial role in the identification and management of dysplasia in patients with ulcerative colitis. The primary objective is the early detection of dysplasia to facilitate timely intervention and continued monitoring. Endoscopic visualization of the colonic mucosa is the cornerstone of surveillance [17]. Historically, dysplasia detection was limited by poor visualization with standard white-light endoscopy, raising concerns about the potential for missed dysplastic lesions. As a result, guidelines incorporating extensive non-targeted (random) biopsies were developed to improve detection rates. These guidelines recommended taking 4 adequately spaced biopsies at 10 cm intervals, yielding a minimum of 33 biopsies [24,25]. During this time, the detection of dysplasia, regardless of grade, generally was considered an indication for immediate colectomy. This approach was driven by the perceived high risk of synchronous or metachronous colorectal cancer and the limitations of available surveillance techniques. This practice was supported by studies, including a 1994 meta-analysis that demonstrated a high risk of CRC if dysplasia was detected during surveillance colonoscopy. Among patients who underwent colectomies, 19% with LGD and 42% with HGD on colonoscopy had CRC [26]. This research, along with other studies, guided clinical practice and reinforced surgery as the standard of care for UC-associated dysplasia during a period dominated by standard white-light endoscopy, less-advanced endoscopic resection capabilities, and less-effective medical therapies.

Over the past two decades, major advances in endoscopic imaging, resection techniques, and medical management have fundamentally changed the surveillance landscape. The adoption of high-definition white-light endoscopy, chromoendoscopy, and enhanced imaging modalities has significantly improved dysplasia detection and lesion characterization. Consequently, endoscopic surveillance has become the cornerstone of management for UC-associated dysplasia, leading to a decline in the recommendation for total proctocolectomy in treating early neoplastic lesions [27]. Professional societies first started publishing formal guidelines on surveillance practices and subsequent management decisions in the early 2000s. The latest evidence-based guidelines were published by SCENIC (Surveillance for Colorectal Endoscopic Neoplasia Detection and Management in Inflammatory Bowel Disease Patients: International Consensus Recommendations) in 2015, the American Gastroenterology Association (AGA) in 2021, the British Society of Gastroenterology (BSG) in 2025, and the European Crohn’s and Colitis Organisation (ECCO) in 2023 [25].

Although society guidelines exhibit some heterogeneity, there is a broad consensus regarding key screening principles. Most guidelines recommend initiating routine dysplasia surveillance 8–10 years after UC diagnosis, reflecting the increased risk associated with longer disease duration. An important exception is that patients with concomitant PSC should undergo immediate surveillance upon PSC diagnosis and annual screening thereafter, given their approximately four-to-five-fold increased risk of CRC [20,25,28,29]. Furthermore, most society guidelines agree that the surveillance follow-up should range from 1 to 5 years and be tailored to individual risk factors, such as disease extent, family history of CRC, disease severity, prior examination findings (including prior dysplastic lesions), and primary sclerosing cholangitis, among others [2,24,28,29]. A comparison of the latest societal guidelines on specific recommendations is summarized in Table 1 and Table 2 [24,30,31,32,33,34].

### 4.2. Terminology

**Figure 2:**
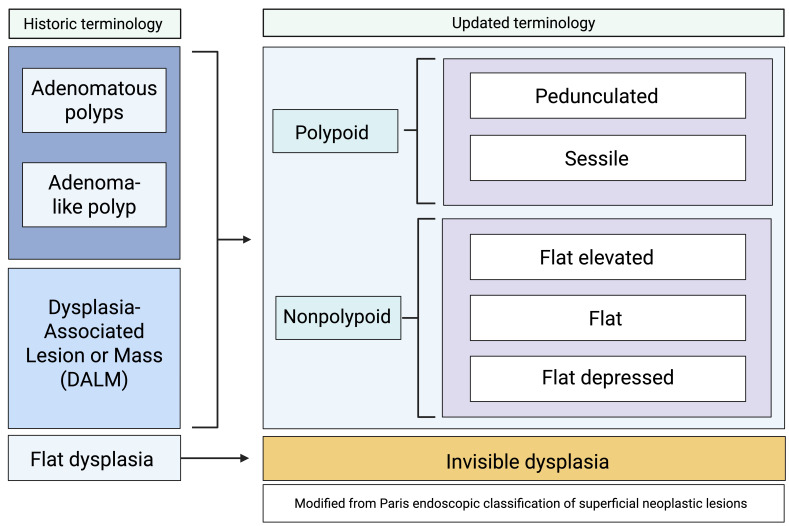
This depicts the nomenclature change. Modified from Paris Classification in 2015. Adapted from the AGA Clinical Practice Update on Endoscopic Surveillance and Management of Colorectal Dysplasia in Inflammatory Bowel Diseases. (Murthy 1). Created in BioRender. Adrienne L. Vickers. (2025) https://app.biorender.com/illustrations/6953f8debe530ba248ed0aad (accessed on 30 December 2025).

Previously, precancerous lesions in individuals with colonic IBD were described using terms such as dysplastic-associated lesion or mass (DALM), adenomatous polyp, adenoma-like mass, or flat dysplasia. Over the past decade, there has been a shift in terminology aimed at standardizing the classification and reporting of IBD-related dysplasia. The current nomenclature is a modified version of the Paris classification, which was recommended in 2015 by the International Surveillance for Colorectal Endoscopic Neoplasia Detection and Management in Inflammatory Bowel Disease Patients consortium [27,34]. Dysplasia is classified as either visible or invisible [25]. Visible dysplasia refers to dysplasia identified through targeted biopsies of a visualized lesion. It can be further divided into polypoid, which is ≥2.5 mm above the mucosa (either pedunculated or sessile), or nonpolypoid < 2.5 mm (flat, flat elevated, or flat depressed) [26,27,34]. Invisible dysplasia, once called flat dysplasia, is dysplasia discovered via non-targeted (random) biopsies without an associated visible lesion [24,25,34]. Additional descriptive features include ulceration and distinction between indistinct and distinct borders (Figure 2).

### 4.3. Surveillance Modalities

Several surveillance modalities exist for the detection of dysplasia in ulcerative colitis. Most current guidelines recommend high-definition white-light endoscopy over standard white-light endoscopy [2,25]. High-definition endoscopes, utilizing a 1080p system, provide significantly higher-resolution images compared with standard-definition endoscopes, which employ a 480p system. This enhanced resolution facilitates superior visualization, enabling endoscopists to observe finer details, subtle mucosal abnormalities, and improved delineation of lesion margins [35]. A retrospective study comparing imaging modalities demonstrated dysplasia detection in twice as many patients undergoing high-definition colonoscopy compared to those examined with standard-definition colonoscopy [36].

Another major advance in surveillance has been the development and adoption of dye chromoendoscopy (DCE). This technique involves the application of dye, most commonly methylene blue or indigo carmine, to the colonic mucosa to better visualize lesions by improving contrast and enhancing surface architecture [34]. Absorptive dyes such as methylene blue are readily taken up by non-inflamed epithelial cells and are poorly taken up by intraepithelial neoplasia and areas of active inflammation, acting as a contrast stain and producing detailed images of the cellular surface. Whereas non-absorptive dyes like indigo carmine seep and collect between mucosal grooves and crevices, highlighting the contours of the mucosal surface [37,38]. Overall, this allows the endoscopist to better distinguish areas of abnormal mucosa from normal mucosa, facilitating targeted biopsies and endoscopic resection of visible lesions [38]. Both the SCENIC and AGA guidelines recommend DCE with HD-endoscopy for surveillance in all patients with colonic IBD when available and performed by an appropriately trained endoscopist [24,34]. Furthermore, there is broad agreement among societal guidelines that, when using standard white-light endoscopy, dye chromoendoscopy should be used to improve dysplasia detection [34]. Supporting these recommendations, a systematic review with meta-analysis examining 6 randomized controlled trials (RCTs) and 5 prospective studies showed that DCE detected more patients with dysplasia than conventional white-light endoscopy [39].

The development of virtual chromoendoscopy (VCE) marks another significant advancement in endoscopic surveillance. VCE encompasses a group of technologies that, similar to traditional chromoendoscopy, enhance mucosal visualization but do so without the use of topical dye. These modalities include narrow-band imaging, I-scan, and Fuji Intelligent Color Enhancement. Narrow-band imaging (NBI) uses optical filters to apply narrow-wavelength light spectra to highlight the mucosal surface and enhance visualization of tissue microvasculature [24,37]. I-scan and Fuji Intelligent Color Enhancement are post-imaging processing techniques that digitally emphasize the vasculature and landscape of abnormal mucosa. Although VCE is generally used as an adjunct and not alone, its role in dysplasia surveillance is evolving. The 2015 SCENIC guidelines state that NBI alone should not replace white-light endoscopy because there is insufficient evidence of benefit [34]. However, the more recent 2021 AGA guidelines recognize that VCE is a suitable alternative to dye chromoendoscopy for detecting dysplasia when used in conjunction with high-definition colonoscopy. This is supported by recent studies demonstrating comparable dysplasia detection rates between HD-NBI with DCE and HD I-scan with DCE [40,41]. Additionally, a meta-analysis of 11 randomized controlled trials found that VCE achieved similar dysplasia detection rates to HD white-light endoscopy and dye chromoendoscopy on a per-patient basis [40]. It is important to note that the choice of imaging modality depends on the endoscopist’s resources and training. VCE is a suitable alternative when used by a trained endoscopist; likewise, if DCE is unavailable, HD-WLE is sufficient. With these advancements, the 45-year-old female patient referenced above, residing in an urban area, would undergo surveillance at her hospital with HD-WLE with DCE instead of standard WLE.

## 5. Management

During a colonoscopy, biopsies can be targeted, nontargeted, or random. As mentioned above, in the past, it was common practice to perform at least 33 random biopsies throughout the colon. This approach was popular due to concerns about missing lesions and the presence of synchronous and metachronous neoplasia in areas of flat dysplasia [24]. Today, with improved visualization of the colon, there are ongoing discussions about whether this is still necessary [25]. AGA guidelines recommend targeted biopsies using white light with DCE or VCE, but if these tools are unavailable and in areas of colitis, they still suggest performing random biopsies [24]. SCENIC guidelines do not provide a specific recommendation but question whether nontargeted biopsies are necessary [34]. This remains an evolving topic, with many guidelines lacking consensus, and updates are being made as new research is published and evaluated.

The management of dysplasia depends on (1) the histology of the lesion, (2) the visibility of the lesion and its borders, (3) the ability to perform complete endoscopic resection of the lesion, (4) the number of dysplastic lesions, and (5) the location of the dysplasia [24,25,34]. In general, the main techniques for endoscopic resection are snare polypectomy, with more advanced techniques such as endoscopic mucosal resection (EMR), endoscopic submucosal dissection (ESD), and endoscopic full-thickness resection (EFTR). A lesion is considered endoscopically resectable if the borders of the lesion are clearly visible, it appears fully excised upon visual inspection after resection, histological examination indicates complete removal, and biopsies taken near the lesion show no dysplasia [34].

With advances in endoscopic technique, endoscopic resection is now considered effective and preferred for clearly defined lesions without signs of invasive carcinoma or submucosal fibrosis [42,43,44]. Although the European Society of Gastrointestinal Endoscopy (ESGE) guidelines addressing endoscopic management of colonic lesions have not been tested in UC patients, they provide a framework for these patients. Per the ESGE guidelines, hot snare polypectomy is recommended for pedunculated polyps with stalks [45]. Superficial sessile or flat lesions < 10 mm should be removed by cold snare polypectomy (CSP). Hot snare polypectomy is also recommended for intermediate (≥10–19 mm) sessile or flat lesions without concern for submucosal invasion. En bloc endoscopic mucosal resection, when feasible, is recommended for large flat lesions (≥20 mm). If feasibility or safety is a concern, piecemeal resection rather than en bloc resection can be used, with the goal of removing the lesion with as few fragments as possible. Endoscopic submucosal dissection should be considered for lesions with high suspicion of superficial submucosal invasion [45,46]. UC patients have specific endoscopic challenges compared to the general population due to more frequent scarring and submucosal fibrosis, chronic inflammation, and ill-defined margins, making resection more technically demanding and increasing the risk of complications [46]. When there is no evidence of multifocal or invisible dysplasia elsewhere, ECCO states that polypoid dysplastic lesions and non-polypoid dysplastic lesions ≤2 cm without signs of invasion can be treated by standard polypectomy and EMR or ESD, respectively, by a trained endoscopist [32]. The specific features of the two endoscopic techniques, EMR and ESD, are summarized in Table 3.

Over the past decade, there has been growing evidence supporting endoscopic resection of visible dysplasia in IBD patients, with studies showing that EMR and ESD are both safe and feasible. A recent meta-analysis by Malik et al., evaluating the efficacy and safety of ESD in IBD patients, included 12 studies and reported pooled en bloc, R0, and curative rates of 92.5% (87.9–95.4%), 81.5% (72.5–88%), and 48.9% (32.1–65.9%), respectively. The reported pooled local recurrence, bleeding, and perforation rates were 3.9% (2–7.5%), 7.7% (4.5–13%), and 5.3% (3.1–8.9%), respectively [48]. Another systematic review by Manta et al. focused on endoscopic outcomes of ESD for visible dysplasia in patients with UC and found that, for 216 lesions, the pooled en bloc and R0 resection rates were 88.4% (83.5–92%) and 78.2% (72.3–83.2%), respectively [49]. Similarly, a combination of multicenter and single-center retrospective and prospective studies evaluating the outcomes, safety, and efficacy of endoscopic resection of IBD-associated neoplasia reported comparable results [49,50,51,52,53,54,55,56,57,58,59,60,61,62]. While the majority of these studies reviewed IBD patients, others focused specifically on UC. The techniques studied were heterogeneous, including ESD or a combination of EMR, ESD, or hybrid ESD. Overall, these studies demonstrated high rates of complete, R0, and en bloc resection, with correspondingly low rates of adverse events. The key findings of these studies are summarized in Table 4.

**Figure 3:**
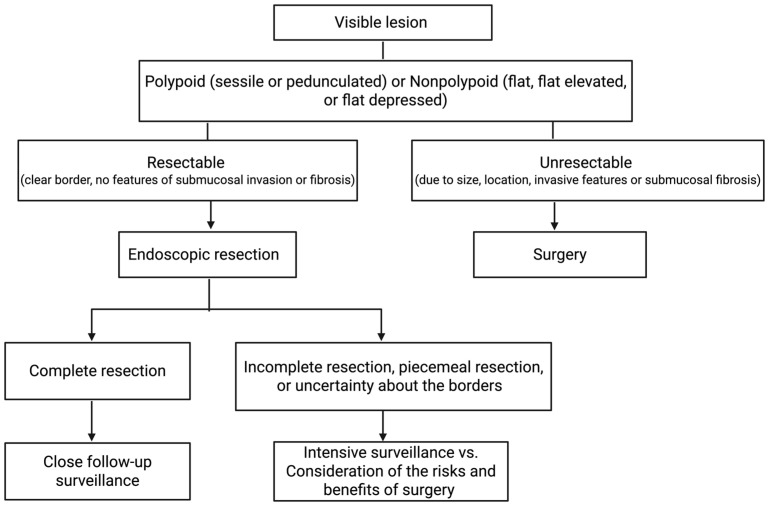
Visible dysplasia suggested treatment algorithm. Adapted from the AGA Clinical Practice Update on Endoscopic Surveillance and Management of Colorectal Dysplasia in Inflammatory Bowel Diseases [20,21]. Created in BioRender. Adrienne L. Vickers. (2025) https://app.biorender.com/illustrations/695439ac6e65957333d11fcd?slideId=11b1fd98-8da9-427e-8799-0d102b16497c (accessed on 30 December 2025).

For visible dysplasia with clear borders, no invasive features, and no submucosal fibrosis, endoscopic resection without colectomy and only follow-up surveillance is acceptable and is not associated with a high risk of CRC [24,25,34]. A meta-analysis of 10 studies with 376 patients showed that after an average of 54 months, only 2.4% developed CRC following endoscopic removal of polypoid dysplasia in IBD [63]. Given the improved visualization using HD endoscopes, the prior recommendation to sample normal mucosa surrounding the lesion to rule out residual dysplasia and confirm complete resection is no longer necessary. This recommendation is based on several studies showing a low incidence of residual dysplasia in the surrounding mucosa [24,64,65]. For nonpolypoid lesions, which may exhibit less distinct borders, or any visible lesion that is excessively large (>2 cm), highly complex, or irregular, advanced endoscopic techniques such as EMR, ESD, or EFTR will most likely be necessary, with close follow-up [24,66]. In cases of complete resection, patients should be monitored with close follow-up, while in cases of piecemeal resection, or uncertainty about borders or the completeness of resection due to the lesion’s complexity, size, or appearance, the risks and benefits of intensive surveillance versus surgery need to be considered depending on the patient factors, histology, and the presence of multifocal disease (Figure 3) [24,25].

**Figure 4:**
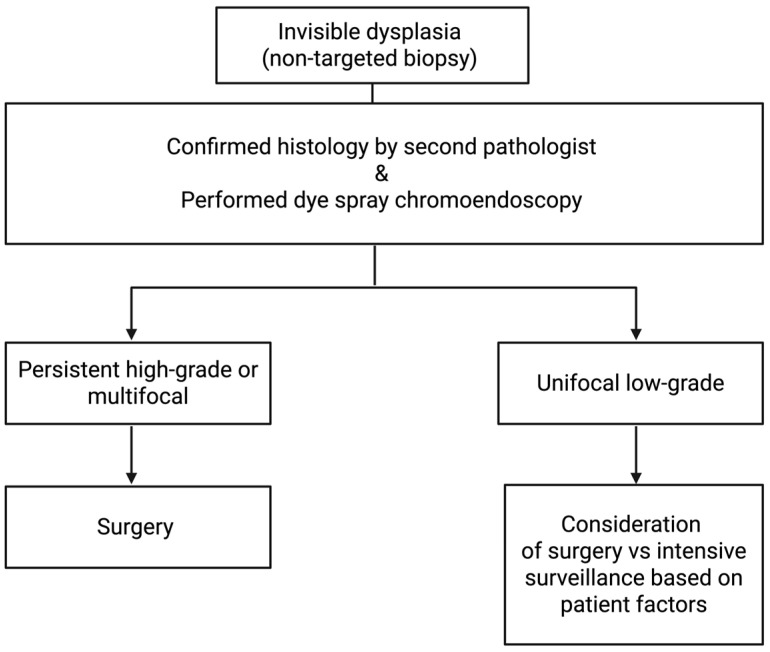
Invisible dysplasia suggested treatment algorithm. Adapted from AGA Clinical Practice Update on Endoscopic Surveillance and Management of Colorectal Dysplasia in Inflammatory Bowel Diseases [20]. Created in BioRender. Adrienne L. Vickers. (2025) https://app.biorender.com/illustrations/6954345630ae2a25ace11617?slideId=8d2343bc-9f09-4b21-9c27-f687f51d813c (accessed on 30 December 2025).

For invisible dysplasia identified during examination and confirmed by a second pathologist, a repeat DCE with HD white-light endoscopy by a skilled endoscopist should be performed for targeted resection [24,34]. Management of persistent, invisible dysplasia varies depending on its grade. Persistent high-grade dysplasia warrants consideration of surgery. The management of persistent low-grade dysplasia is less definitive (Figure 4). If persistent unifocal low-grade dysplasia is found on repeat endoscopy, the choice between intensive surveillance and colectomy depends on individual patient factors [24,25]. Factors to consider include adherence to the treatment plan, PSC, family history of CRC, disease duration, and comorbidities. If visible dysplasia is unresectable, or if signs of invasive carcinoma, multifocal invisible dysplasia, or high-grade invisible dysplasia are present, then surgery should be considered [24]. When high-grade dysplasia and multifocal low-grade dysplasia are detected within visible lesions, surgery is often recommended due to the concern for synchronous and metachronous cancers [67]. Colonoscopy revealed LGD in a flat mucosal area in the 45-year-old female patient referred to above. She was referred to a colorectal surgeon to discuss total proctocolectomy, but today would likely undergo intensive surveillance as part of a more patient-centered treatment approach that considers all risk factors.

## 6. Surgery

Surgery continues to play an important role in managing colitis-associated dysplasia, but it is now generally considered a last resort. For some patients, surgery is clearly the treatment of choice, but for many, the decision is complex and made after careful discussion and weighing of the risks and benefits. The role of surgery has evolved from cancer prevention by performing prophylactic proctocolectomies to being used as the treatment of choice once dysplasia is detected during surveillance, to being used as management for a subset of patients [68]. Total proctocolectomy is the standard surgical recommendation for neoplasia in UC, as it removes nearly all potential dysplastic colonic tissue, thereby lowering cancer risk. Although less common, segmental and total colectomies are occasionally performed in select patients without active inflammation [25]. These select patients are typically elderly, with no active disease, limited disease duration, and localized dysplasia, cancer, or colonic stenosis who are frail and not great surgical candidates [69,70]. There are several reconstruction approaches associated with total proctocolectomy, including ileal pouch-anal anastomosis (IPAA) or an end ileostomy, and less commonly, a continent ileostomy, known as a Koch pouch [25].

The IPAA was first described in 1978 by Parks and Nicholls of St. Mark’s Hospital and has since become a standard surgical procedure. It is typically performed in one stage, two stages (proctocolectomy, IPAA, and loop ileostomy, which is later reversed), modified two stages (subtotal colectomy with end ileostomy, completion proctectomy with IPAA), or three stages (subtotal colectomy with end ileostomy, completion proctectomy with IPAA and loop ileostomy, and reversal of the loop ileostomy), depending on the patient’s preference for restoring intestinal continuity [71,72]. This operation involves a near-complete proctocolectomy followed by either hand-sewing or stapling the ileal pouch to the anal canal. The stapled method uses a double-staple technique, usually with a stapler to transect the rectum and pouch, and an end-to-end anastomosis stapler to create the anal anastomosis. The hand-sewn method involves transanal mucosectomy followed by hand-sewing of the ileal pouch to the anal canal [72]. Originally designed to remove all large intestinal mucosa, the procedure evolved into a less technically challenging technique associated with fewer complications, leaving a small amount of rectal mucosa due to its challenging nature, inferior function, and complications [73]. Although the procedure overall significantly reduces the risk of developing UC-related cancer, it does not completely eliminate it due to the small amount of remaining rectal mucosa at the anal transitional zone. In a review article that examined 3203 UC patients who underwent IPAA, the cumulative incidence of neoplasia increased from 0.9%, 1.3%, 1.9%, 4.2% and 5.1% at 5, 10,15, 20, and 25 years, respectively, indicating that while the risk is small, it is not completely eliminated [74]. Routine surveillance of the pouch is therefore important and recommended, in accordance with various society guidelines. The AGA, ASGE, BSG, and ECCO guidelines recommend at least annual pouch surveillance for those at high risk of developing colorectal dysplasia, such as individuals with PSC, a history of colorectal cancer or dysplasia, persistent pouchitis, or atrophic and inflamed mucosa [24,31,32,75]. If dysplasia develops, there are no official guidelines specifically for pouch neoplasia. It most commonly occurs in the anal transition zone but can also develop within the pouch or residual rectal cuff [25]. The management of pouch neoplasia is individualized and should be discussed within a multidisciplinary tumor or IBD board.

## 7. Future Directions

Advances in research and technology are deepening our understanding of ulcerative colitis, opening new avenues for managing dysplasia in UC. Key areas of ongoing development include AI-assisted detection, molecular markers, chemoprevention strategies, and emerging endoscopic techniques and technologies.

### 7.1. Artificial Intelligence

The utilization of artificial intelligence (AI) in medicine is expanding, with its integration into numerous innovative applications. Artificial intelligence encompasses machine learning techniques designed to emulate human cognition. It has been investigated as a supportive tool for traditional colonoscopy to address certain limitations in current neoplasia surveillance strategies, including variability in disease presentation and associated risks, imperfect endoscopic techniques, and susceptibility to interobserver variability in lesion assessment [76]. Image recognition is one method by which these systems are trained to identify pathology endoscopically [77]. Convolutional neural networks (CNNs)-based computer-aided detection (CADe) systems are AI tools developed to assist in polyp detection [78].

The most robust and validated AI systems have been developed using datasets derived from sporadic colorectal polyps rather than IBD-associated dysplasia. Several evidence-based studies have demonstrated that AI-assisted colonoscopies improve adenoma detection rates (ADR) in patients. A 2023 systematic review and meta-analysis of 21 RCTs involving 18,232 patients reported a higher ADR in the CADe group (44.0%) compared with standard colonoscopy (35.9%), along with a 55% relative reduction in miss rate [78]. In a more comprehensive systematic review and meta-analysis by Soleymanjahi et al. in 2024, 43 RCTs comparing CADe with conventional colonoscopy were evaluated [79]. Overall, results showed an increase in the average number of adenomas per colonoscopy and ADR, but no difference in the detection of advanced colorectal neoplasia. The study reported polyp detection rates of 56.1% for CADe and 47.9% for standard colonoscopy, and ADRs of 44.8% for CADe and 37.4% for standard colonoscopy [79]. These findings support the clinical utility of AI in enhancing polyp detection in the general population.

In contrast to CRC screening in the general population, the application of AI in the detection of IBD-associated dysplasia remains in early stages of development. Endoscopic surveillance in IBD patients presents unique challenges, including active disease, inflammatory pseudopolyps, and invisible dysplasia. Early clinical investigations have largely involved adapting CADe systems initially developed for non-IBD populations. In 2020, in Japan, one of the first reported cases of a CADe system (EndoBrain technology), originally designed for non-IBD screening, was used for IBD screening in a 72-year-old patient with an 18-year history of UC, and while using HD-WLE and this AI detection system, 2 lesions of LGD were identified in the sigmoid colon [80]. However, its broader clinical applicability remains uncertain. A 2024 single-center retrospective cohort study conducted in Israel assessed the utility of a generic CADe system (GI Genius) in IBD patients undergoing dysplasia surveillance. The study found no significant improvement in neoplasia detection rates, highlighting the limitations of applying non-IBD-trained CADe systems to an IBD population [81]. To address these limitations, recent efforts have focused on developing IBD-specific CADe systems. A study by Yamamoto et al. developed a CNN-based AI system to identify IBD neoplasia. This system was trained using 862 IBD neoplasia images and tasked with classifying images into two groups: “adenocarcinoma/high-grade dysplasia” and “low-grade dysplasia/sporadic adenoma/normal mucosa.” The AI system outperformed expert endoscopists (sensitivity: 60.5%, specificity: 88.0%, accuracy: 77.8%) and non-expert endoscopists (sensitivity: 70.5%, specificity: 78.8%, accuracy: 75.8%), achieving a sensitivity of 72.5%, specificity of 82.9%, and overall accuracy of 79.0% [82]. Similarly, a 2024 study by Abdelrahim et al. developed and validated a system for lesion detection and characterization specifically for IBD patients, using over 18,000 images for training. Image-based validation involved 478 images, while real-time clinical validation included 30 consecutive patients. The reported lesion detection rate was 90.4% [83]. AI has the potential to enhance ulcerative colitis-associated dysplasia surveillance by improving the detection and characterization of lesions.

Although AI shows considerable promise for improving dysplasia detection in ulcerative colitis, current evidence remains limited compared with that in the general population. Further refinement of AI systems with larger, prospective, IBD-specific validation trials and RCTs is needed. Additionally, technical and practical challenges must be addressed before clinical integration can be achieved [84].

### 7.2. Molecular Endoscopy

Molecular endoscopy is another area for potential advancement in the detection of colitis-associated neoplasia. Molecular endoscopy combines optical imaging with disease-specific fluorescent probes to improve overall lesion detection by using molecular markers such as peptides, small molecules, or fluorescent antibodies that selectively bind to targets expressing gastrointestinal disease, allowing real-time visualization of pathological changes. The main molecular imaging modalities are confocal laser endomicroscopy, fluorescence molecular endoscopy, and endocytoscopy [85].

Confocal laser endomicroscopy (CLE), introduced in the early 2000s, is a technology that integrates laser scanning microscopy into an endoscope or probe, providing high-resolution, histology-level in vivo imaging, enabling up to 1000-fold magnification [85,86]. The use of topical or intravenous fluorescent dyes with the CLE probe enhances visualization of the epithelium, introducing the concept of an in vivo “optical biopsy” [87]. CLE has been primarily utilized to assess UC activity and inflammation and has also shown promise in predicting clinical response in Crohn’s disease [88,89]. Recent studies have examined the role of CLE in detecting dysplasia, with promising results [89,90,91,92,93]. A randomized trial of 161 patients with UC comparing conventional colonoscopy with chromoendoscopy and CLE identified 4.75 more dysplastic lesions and required 50% fewer biopsies when using chromoendoscopy with CLE [90]. Another RCT with 162 UC patients, evaluating chromoendoscopy with CLE versus conventional colonoscopy for dysplasia surveillance, did not find improved neoplasia detection with CLE. Despite this, it reported a positive outcome of a decrease in the number of biopsies performed [92]. Although CLE shows potential for in vivo histology, increased procedural time, equipment malfunction and failure, cost, and ambiguous and unclear results regarding superiority are barriers to clinical implementation [89].

Fluorescence molecular endoscopy uses targeted fluorescent probes to distinguish between normal and diseased tissue [85]. This modality is mainly used in esophageal cancer, Barrett’s esophagus, and gastric cancer. More recently, probes have been developed to identify active inflammation and predict response to biologic therapies in IBD patients. There has also been promising early research on the detection of colonic neoplasia, which would be helpful for using disease-specific biomarkers rather than just morphologic changes [85]. A study evaluated fluorescent endoscopy using a cathepsin activity-based molecular probe to detect colitis-induced dysplasia in mice with induced colitis [94]. Cathepsins are lysosomal cysteine proteases that contribute to the proteolytic network in tumor microenvironments [95]. The results suggest that cathepsin activity can be used to distinguish dysplastic tissue from benign areas of chronic inflammation [94]. Further studies are needed to explore the potential of this technology in this area.

Endocytoscopy (EC) is an advanced imaging modality that provides ultra-high magnification (450×–1400×), enabling microscopic observation at the cellular level. This technique combines an optical endoscope with a mucolytic agent (e.g., N-acetylcysteine) to enhance the penetration of a topical contrast-absorbing agent, such as methylene blue [89,96]. EC uses the principle of contact light microscopy and was developed to bridge the diagnostic gap between endoscopy and histology [96,97]. It allows real-time visualization of cellular structures, including inflammatory cells and nuclear details, of the superficial epithelial layer during endoscopic procedures [98]. EC has been useful for assessing inflammation and has also been shown to play a role in the detection of colitis-related dysplasia. A recent pilot study found that EC could effectively predict CAN based on nuclear irregularities [99]. This single-center retrospective study evaluated EC with pit pattern analysis to differentiate UCAN from non-neoplastic lesions in UC patients. It analyzed 103 lesions in 62 patients and found that EC exhibited higher specificity and accuracy for diagnosing UCAN than classical pit pattern analysis [99]. While EC shows promise, limitations include inflammation-induced vascular changes that can mimic malignancy and potentially lead to misclassification, as well as the high cost and associated learning curve [89,96]. Overall, further studies are needed to validate the use of EC in IBD surveillance.

### 7.3. Molecular Biomarkers and Chemoprevention

Molecular biomarkers for cancer associated with inflammatory bowel disease are another promising area for earlier, more accurate, and less invasive detection. A wide range of emerging biomarkers exists, including genetic, epigenetic, immune-mediated, proteomic, and RNA-based markers. Progress has been made in identifying novel biomarkers for UCAN, but their clinical application remains to be determined [100].

Chemoprevention remains an important area of research for dysplasia in ulcerative colitis. Potential agents are aminosalicylates, immunomodulators, ursodeoxycholic acid, and anti-TNF drugs. Aminosalicylates, including sulfasalazine and mesalamine (5-ASA), reduce mucosal inflammation topically [101]. In population-based studies, there was no reduction in the risk of CRC, whereas in clinical studies, there was evidence of risk reduction [101]. A meta-analysis investigating the relationship of 5-ASA use and colorectal neoplasia risk in IBD patients examined 31 observational studies and found a chemopreventive role and protection for UC patients, reporting a pooled 43% decrease in the risk of developing colorectal neoplasia [102]. The reduction in colorectal neoplasia associated with 5-ASA in IBD patients was 50% among UC patients [102]. There are varied studies with heterogeneous findings for thiopurines, biologics, acetylsalicylic acid, nonsteroidal anti-inflammatory drugs, ursodeoxycholic acid, and statins. Future surveillance studies need to be conducted to assess the effects of chemoprevention with longer follow-up, across larger populations, and at different dosages to evaluate the impact of drugs on CRC risk.

## 8. Limitations

This review has several limitations. The discussion of management provides a general overview of societal guidelines, but does not go into each guideline in detail or examine their differences. Secondly, the screening and management are directed more toward highly industrialized countries, understanding that, due to limited access to advanced therapies, certain resource-limited regions may have different approaches. Lastly, the literature search may have missed some relevant articles.

## 9. Conclusions

Overall, this field continues to advance, driven by innovations not only in endoscopic techniques but also in technology transforming the surveillance and management of colitis-associated dysplasia. Treatment strategies have shifted from total proctocolectomy to endoscopic resection of lesions and close follow-up. These developments, along with improved risk stratification, have led to more individualized care for patients with ulcerative colitis-associated dysplasia.

## Figures and Tables

**Table 1:** Comparison of societies’ recommendations on colonoscopy initiation, surveillance intervals, and imaging modality.

Society	Initiate	Surveillance	Imaging Modality
ACG 2019 [30]	8 years after the onset of symptoms in all patients with IBD; from diagnosis if PSC	Every 1–3 years based on risk factors for CRC and findings on previous colonoscopy. Annually if PSC.	Standard-definition endoscope use DCE; if HD-WLE uses NBI or DCE
AGA 2021 [24]	8–10 years after disease onset in all patients with colonic IBD, from diagno-sis if PSC	Every 1–5 years based on risk factors for CRC: burden of colonic inflammation, FH of CRC, PSC, history of dysplasia, and quality and frequency of prior exams.	DCE should be performed over all other modalities if available. If standard-definition endoscope use DCE; VCE is a suitable alternative to DCE when using HD. HD-WLE is preferable over standard definition endoscope
BSG 2025 [31]	8 years after onset of symptoms; from diagnosis if PSC *	Very low risk: q10 years (no additional risk factors or calculated risk of CRC at 5 years close to general population); Small risk: q3 years (mild active inflammation, extensive disease, post-inflammatory polyps, FH of CRC in 1st degree relative, or calculated small risk of advanced CRC at 5 years); Moderate risk: q1 year (moderate active inflammation, stricture/dysplasia in last 5 years, PSC *, or calculated moderate risk of advanced CRC at 5 years); Large risk: consider colectomy (severe active inflammation or calculated large risk of CRC at 5 years)	HD-DCE over all other modalities if available; HD-WLE over standard definition endoscope; No GRADE recommendation for VCE
ECCO 2023 [32]	8 years after onset of symptoms; from diagnosis if PSC *	Low risk: q5 years (colitis affecting < 50% of colon, minimal inflammation); Intermediate risk: q2-3 years (mild to moderate inflamma-tion, CRC in 1st degree family member > 50 years); High risk: q1 year (extensive colitis with severe active inflammation, CRC in a first-degree relative ≤50 years of age, stricture/dysplasia, PSC) *	No specific recommendation
NCCN 2025 [33]	8 years after the onset of symptoms, from diagnosis if PSC	Surveillance in 2 to 5 years if low risk for CRC. High risk (PSC, active inflammation, FH of CRC at < 50 years of age) surveillance in 1 year.	HD-WLE recommended with targeted and random biopsies or DCE or HD-VCE with targeted biopsy; if using a standard definition endoscope use DCE If HD-WLE or DCE are unavailable refer to another institution
SCENIC 2015 [34]	No specific recommendation	Repeat every 3–6 months after visible lesions resected; smaller lesions can repeat in 1 year	When using WLE, use HD over standard definition. When using standard definition or HD, use CE over white light. NBI is not suggested in place of white light or CE

ACG, American College of Gastroenterology; IBD, inflammatory bowel disease; PSC, primary sclerosing cholangitis; CRC, colorectal cancer; FH; family history, DCE, dye chromoendoscopy; HD-WLE, high-definition white light endoscopy; NBI, narrow-band imaging; AGA, American Gastroenterological Association; VCE, virtual chromoendoscopy; BSG, British Society of Gastroenterology; ECCO, European Crohn’s and Colitis Organization; NCCN, National Comprehensive Cancer Network; SCENIC, Surveillance for Colorectal Endoscopy Neoplasia Detection and Management in Inflammatory Bowel Disease Patients: International Consensus Recommendations; CE, chromoendoscopy. * Including post-liver transplant.

**Table 2:** Comparison of societies’ recommendations on dysplasia management and follow-up and surveillance of the ileal anal pouch.

Society	Dysplasia Management	Dysplasia Follow-Up	Ileal-Anal Pouch
ACG 2019 [30]	No specific recommendation. If dysplasia is not resectable or is multifocal, refer for proctocolectomy	When dysplasia is resected, subsequent surveillance colonoscopy performed at shortened intervals. When dysplasia not resectable or is multifocal, refer for proctocolectomy.	No specific recommendation
AGA 2021 [24]	Clear demarcated visible lesions * undergo endoscopic resection, if resectability is in question refer to specialized endoscopist or IBD center; Unresectable visible dysplasia or invisible multifocal should prompt colectomy	Invisible dysplasia should prompt repeat colonoscopy use HD-DCE with extensive random biopsies. HGD should prompt colectomy. Resected visible lesions or if histologic dysplasia is not confirmed on a high-quality DCE examination, continue endoscopic surveillance at frequent intervals.	Pouch surveillance at least annually for high risk for colorectal dysplasia (prior CRC or dysplasia, PSC) and moderate to severe pouchitis and/or pre-pouch ileitis; Surveillance for lower risk should be individualized.
BSG 2025 [31]	Adenoma or serrated lesion outside of a colitis affected segment should be managed by sporadic post-polypectomy guidelines; Visible dysplasia consider endoscopic en bloc resection *; ≥ 2 cm nonpolypoid lesions undergo advanced endoscopic en bloc resection; Consider surgery for unresectable lesions or when surveillance is not effective or possible	En bloc resected polypoid LGD < 2 cm follow up annually for 5 years if no recurrence. HGD or LGD with high-risk features (≥2 cm, non-polypoid, multifocal, or piecemeal resection): surveillance after 3–6 months, then annually for 5 years if no recurrence. Invisible dysplasia: repeat colonoscopy with CE and segmental or mapping biopsies in 3–6 months. Persistent invisible HGD or multifocal LGD: consider colectomy. Indefi-nite dysplasia: optimize medical therapy and repeat colonos-copy within 3–6 months, with targeted and segmental map-ping biopsies. Multifocal or invisible dysplasia with high ad-vanced neoplasia risk: consider surgery.	Annual surveillance for patients who had surgery for colitis-associated dysplasia or cancer; Every 1–3 years surveillance for patients with PSC, cuffitis, pouchitis, CD, long duration of UC or FH of CRC in 1st degree relative; No risk factors require no additional surveillance until endoscopic reassessment at 10 years
ECCO 2023 [32]	Polypoid lesions or non-polypoid lesion endoscopic en bloc resection, when no evidence of multifocal or invisible dysplasia elsewhere *. Consider surgery for unresectable dysplastic lesions and evidence of multifocal or invisible dysplasia elsewhere.	Surveillance with DCE or VCE + targeted and random biop-sies every 3 to 6 months for the first year, then annually based on grade of dysplasia. Visible HGD:3 months for the first year, then annually; Non-polypoid LGD: 6 months for the first year, then annually; Polypoid < 1cm or pedunculated LGD: 12months; Non-polypoid large lesion > 2 cm: intensive sur-veillance with DCE or VCE plus targeted and random biop-sies every 3 to 6 months for the first year and then annually; Invisible dysplasia: repeat surveillance with HD-DCE or VCE plus random and target biopsies; Persistent unifocal LGD: consider intensive DCE follow-up; Persistent unifocal HGD: consider colectomy. Indefinite dysplasia: optimize therapy and repeat surveillance with HD-DCE or VCE plus random and targeted biopsies; Multifocal dysplasia LGD or HGD: consider surgery.	Annual surveillance for patients who had surgery for colitis-associated dysplasia or cancer and in patients with PSC.
NCCN 2025 [33]	Resectable visible dysplasia should undergo endoscopic resection. Non-resectable polypoid lesion or mass refer to surgeon with IBD expertise	Low risk resectable lesion (hyperplastic or normal mucosa, no active inflammation, <1 cm LGD) surveillance in 1–3 years; Higher risk resectable lesion (PSC, ≥1 cm LGD, active inflammation, FH of CRC < 50 years of age, HGD) surveillance in 1 year; ≥2 cm LGD, HGD, or piecemeal resection surveillance in 3–6 months; Incomplete endoscopic resection refer to advanced endoscopist or consider referral to surgeon with expertise in IBD; Invisible dysplasia assess with CE if not done and consider referral to surgeon with expertise in IBD	No specific recommendation
SCENIC 2015 [34]	Endoscopic resection of polypoid and nonpolypoid dysplastic lesions if able with surveillance colonoscopy	After removal of endoscopically resectable nonpolypoid and polypoid dysplastic lesions, surveillance colonoscopy rather than colectomy is recommended. Endoscopically invisible dysplasia referral is suggested to an endoscopist with expertise in IBD using HD-chromoendoscopy.	No specific recommendation

CD, Crohn’s disease; UC, ulcerative colitis; HGD, high grade dysplasia; LGD, low grade dysplasia; * Without stigmata of invasive cancer or fibrosis and distinctive border.

**Table 3:** Main technical features of endoscopic mucosal resection (EMR) and endoscopic submucosal dissection (ESD).

Endoscopic mucosal resection	EMR involves the injection of a solution into the submucosal space to separate a mucosal lesion from the underlying muscularis propria. The lesion can then be resected by snare electrosurgery. The submucosal cushion theoretically reduces the risk of thermal or mechanical injury to the underlying muscularis propria [45].
Endoscopic submucosal dissection	ESD involves three steps: Injection of fluid into the submucosa to lift the lesion from the muscle layer. Circumferential cutting of the mucosa surrounding the lesion. Dissection of the connective tissue of the submucosa beneath the lesion. Advantages of this technique include controllability of resected size and shape, en bloc resection in large neoplasms, and resectability of neoplasms with submucosal fibrosis. Thus, this technique can be applied to the resection of complex neoplasms, including large, ulcerative, non-lifting, and recurrent neoplasms. Disadvantages of this technique include its time-consuming nature and a higher risk of bleeding and perforation than EMR [47].

**Table 4:** Study data on outcomes of endoscopic procedures in patients with inflammatory bowel disease (IBD) over the last 10 years.

Author, Publication Year	Country	Design	Condition	No. of Subjects (No. of Lesions)	Procedure	Lesion Size Median Range (Range), mm	En Bloc Resection (%)	R0 Resection (%)
Manta et al. 2021 [49]	Italy	M-C	UC	53 (53)	ESD	34 (20–50)	53 (100)	51 (96.2)
Kasuga et al. 2021 [50]	Japan	S-R	UC	9 (11)	ESD	30 (20–50)	10 (91)	9 (82)
Lightner et al. 2021 [51]	USA	S-R	UC, CD	25 (25)	ESD	30 (22.5–37.5)	23 (88)	23 (88)
Ngamruengphong et al. 2022 [52]	USA	M-R	UC, CD	41 (45)	ESD	30 (23–42)	43 (96)	34 (76)
Iacopini et al. 2015 [53]	Italy Japan	M-P	UC	9 (10)	ESD	15 (10–20)	8 (80)	7 (70)
Gulati et al. 2018 [54]	UK	S-P	UC, CD	15 (15)	EMR, ESD, hESD	48.3 (20–90)	6 (40)	6 (40)
Suzuki et al. 2017 [55]	UK Japan	M-R	UC	32 (32)	ESD	33 (12–73)	29 (91)	23 (79)
Kochhar et al. 2018 [56]	USA	S-P	UC, CD	7 (7)	ESD	40.7 (25–70)	6 (85.7)	NA
Yang et al. 2019 [57]	S. Korea	S-R	UC	25 (25)	ESD	33 (18–60)	14 (93.3)	12 (80)
Matsumoto et al. 2021 [58]	Japan	S-R	UC	7 (12)	ESD	15 (8–20)	10 (83)	8 (67)
Yadav et al. 2019 [59]	USA	S-R	UC, CD	97 (124)	EMR, ESD, hESD	NA	88 (70.9)	NA
Nishio et al. 2021 [60]	Japan	S-R	UC	74 (102)	EMR, ESD	12 ± 9.6 *	97 (95)	88 (86)
Kinoshita et al. 2018 [61]	Japan	M-R	UC	25 (25)	ESD	34.9 ± 17.1 *	25 (100)	19 (76)
Maselli et al. 2025 [62]	Italy Canada Norway	M-R	UC, CD	91 (96)	ESD, hESD	34.8 ± 16.2 *	92 (95.8)	82 (85.4)

S-R, single-center retrospective; M-R, multi-center retrospective; M-P, multi-center prospective; S-P, single-center prospective; M-C, multi-center case series; UC, ulcerative colitis; CD, Crohn’s disease; ESD, endoscopic submucosal dissection; EMR, endoscopic mucosal resection; hESD, hybrid endoscopic submucosal dissection; * Mean ± SD. NA: not available.

## Data Availability

No new data was created for this work.

## Abbreviations

The following abbreviations are used in this manuscript:

UC	Ulcerative Colitis
CRC	Colorectal Cancer
SIR	Standardized incidence ratio
HD	High definition
IBD	Inflammatory bowel disease
CAN	Colitis-associated neoplasia
UCAN	UC-associated neoplasia
LGD	Low-grade dysplasia
HGD	High-grade dysplasia
APC	Adenomatous polyposis coli
PSC	Primary sclerosing cholangitis
WLE	White light endoscopy
SCENIC	Surveillance for colorectal endoscopic neoplasia detection and management in inflammatory bowel disease patients: International Consensus Recommendations
AGA	American Gastroenterology Association
BSG	British Society of Gastroenterology
ECCO	European Crohn’s and Colitis Organization
CE	Chromoendoscopy
DALM	Dysplastic-associated lesion or mass
DCE	Dye chromoendoscopy
RCT	Randomized controlled trial
VCE	Virtual chromoendoscopy
NBI	Narrow-band imaging
EMR	Endoscopic mucosal resection
ESD	Endoscopic submucosal dissection
EFTR	Endoscopic full-thickness resection
ESGE	European Society of Gastrointestinal Endoscopy
CSP	Cold snare polypectomy
IPAA	Ileal pouch-anal anastomosis
AI	Artificial Intelligence
CNNs	Convolutional neural networks
CADe	Computer-aided detection
ADR	Adenoma detection rate
CLE	Confocal laser endomicroscopy
EC	Endocytoscopy
